# Molecular Identification of *Plasmodium falciparum* from Captive Non-Human Primates in the Western Amazon Ecuador

**DOI:** 10.3390/pathogens10070791

**Published:** 2021-06-22

**Authors:** Gabriel Alberto Carrillo Bilbao, Juan-Carlos Navarro, Mutien-Marie Garigliany, Sarah Martin-Solano, Elizabeth Minda, Washington Benítez-Ortiz, Claude Saegerman

**Affiliations:** 1 Instituto de Salud Pública y Zoonosis (CIZ), Universidad Central del Ecuador, Quito 170521, Ecuador; gabriel.carrillobilbao@doct.uliege.be (G.A.C.B.); ssmartin@espe.edu.ec (S.M.-S.); elizabeth.minda@gmail.com (E.M.); wbenitez@uce.edu.ec (W.B.-O.); 2 Research Unit of Epidemiology and Risk Analysis Applied to Veterinary Sciences (UREAR-ULg), Fundamental and Applied Research for Animal and Health (FARAH) Center, Department of Infections and Parasitic Diseases, Faculty of Veterinary Medicine, University of Liège, B-4000 Liège, Belgium; 3 Grupo de Investigación en Enfermedades Emergentes, Ecoepidemiología y Biodiversidad, Facultad de Ciencias de la Salud, Universidad Internacional SEK, Quito 170107, Ecuador; juancarlos.navarro@uisek.edu.ec; 4 Department of Pathology, Fundamental and Applied Research for Animal and Health (FARAH) Center, Liège University, B-4000 Liège, Belgium; mmgarigliany@uliege.be; 5 Department of Animal Pathology, Liège University, B-4000 Liège, Belgium; 6 Grupo de Investigación en Sanidad Animal y Humana (GISAH), Carrera Ingeniería en Biotecnología, Departamento de Ciencias de la Vida y la Agricultura, Universidad de las Fuerzas Armadas—ESPE, Sangolquí 171103, Ecuador

**Keywords:** *Leontocebus lagonotus*, malaria, faecal samples

## Abstract

**Background:** Malaria is a disease caused by hemoparasites of the *Plasmodium* genus. Non-human primates (NHP) are hosts of *Plasmodium* sp. around the world. Several studies have demonstrated that *Plasmodium* sp. emerged from Africa. However, little information is currently available about *Plasmodium falciparum* in the neotropical NHP and even less in Ecuador. Indeed, the objective of our study was to identify by molecular phylogenetic analyses the *Plasmodium* species associated with NHP from the Western Amazon region of Ecuador, and to design a molecular taxonomy protocol to use in the NHP disease ecology. **Methods:** We extracted DNA from faecal samples (n = 26) from nine species of captive (n = 19) and free-ranging (n = 7) NHP, collected from 2011 to 2019 in the Western Amazon region of Ecuador. **Results:** Using a pan-Plasmodium PCR, we obtained one positive sample from an adult female *Leontocebus lagonotus*. A maximum likelihood phylogenetic analysis showed that this sequence unequivocally clustered with *Plasmodium falciparum*. **Conclusions:** The identification of *Plasmodium* sp. in NHP of the Ecuadorian Amazon would be essential to identify their role as potential zoonotic reservoirs, and it is also important to identify their origin in wildlife and their transmission in captive NHP.

## 1. Introduction

Malaria is a disease transmitted by mosquitoes and caused by a hemoparasite of the genus Plasmodium. The protozoan *Plasmodium* spp. belongs to the Apicomplexa groups; they have the ability to parasite cells thanks to a group of organelles called the apical complex. *Plasmodium* inhabits red blood cells and hepatocytes [1]. It causes high fever, anemia, headaches, and diarrhea, among others. This mosquito-borne disease is found in humans and in several vertebrates such as birds [2,3], bats [4], antelopes and reptiles [5], and non-human primates (NHP) [6,7,8,9]. The latter are an important source of infections for *Plasmodium* sp. in humans. In OW (Old World) monkeys, *Plasmodium* sp. has been detected in chimpanzees (*Pan troglodytes*) [10], baboons (*Papio anubis*), Tantalus monkeys (*Chlorocebus aethiops*), red patas monkeys (*Erythrocebus patas*) [6], long-tailed and pig-tailed macaques (*Macaca fascicularis* and *Macaca nemestrina*) [7], and recently in a lemur species (*Propithecus verreauxi)* and *Macaca radiata* [11], to name a few. In the neotropics, there is evidence of natural infection in humans with *Plasmodium brasilianum* in Venezuela [12] and *Plasmodium simium* in Brazil [13]. These two species of Plasmodium naturally infect NHP. *Plasmodium brasilianum* has been identified in at least 49 species of New World (NW) NHP [14,15,16,17,18,19,20] (Table 1). *Plasmodium simium* has been found in less species, around 4 NW NHP species [18,21,22,23]. There are 25 known species of Plasmodium and some of those (*P. vivax*, *P. malariae*, *P. falciparum*, *P. ovale*, *P. knowlesi*) are responsible for human malaria [24,25,26]. *Plasmodium falciparum* is one of the most malignant species of malaria and it originated from human migration into the NW [10,27,28].

Several genes are used for molecular identification and phylogenetic studies of *Plasmodium* species [29,30]. However, the small subunit ribosomal RNA gene is widely used for molecular characterization and phylogenetic studies [31,32,33]. Indeed, it has both highly conserved and very variable domains. This gene was used to study the phylogenetic relationships [34,35,36] and host specificity of *Plasmodium* sp. [19]. More than 49 species of NW monkeys are known to be infected with *Plasmodium* sp. [37,38]. In Ecuador, only a few studies on *Plasmodium* sp. were realized [39]. Avian Plasmodia were studied in the Galapagos [40,41]. In humans, several studies with molecular markers have identified population origins of *P. falciparum* in the Northwest of Ecuador [42,43], but only one study yielded sequence information [44]. The aim of this study was to monitor *Plasmodium* sp. in the Amazon region of Ecuador, to identify potential zoonotic reservoirs, and to identify the origins of malaria parasites in wildlife and potential human–monkey transmission with captive NHP.

**Table 1:** *Plasmodium* species found in neotropical non-human primates.

Host	Location	Plasmodium Species	Sampling (Invasive Non-Invasive)	Detection Methods	References
*Alouatta seniculus*.	Brazil	*Plasmodium* sp.	Invasive	Conventional microscopy (GIEMSA) PCR	[17]
*Alouatta caraya* *Alouatta guariba clamitans* *Alouatta seniculus macconnelli* *Sapajus apella*	Brazil French Guiana	*Plasmodium vivax*	Invasive	Microscopy Enzyme-linked Immunosorbent assay IFA ELISA PCR Real-time PCR	[22,45,46]
*Alouatta* sp. *Alouatta seniculus* *Alouatta seniculus straminea* *Alouatta caraya* *Alouatta guariba clamitans* *Alouatta guariba guariba* *Aotus nigriceps* *Alouatta g. clamitans* *Ateles* sp. *Ateles belzebuth* *Ateles chamek* *Ateles paniscus* *Aotus nigriceps* *Bracytheles arachnoides* *Cacajao calvus* *Cacajao rubicundus* *Callicebus bruneus* *Callicebus dubuis* *Callicebus moloch* *Callicebus personatus* *Callicebus torquatus* *Callithrix geoffroyi* *Cebus* sp. *Chiropotes albinasus* *Chiropotes chiropotes* *Chiropotus* sp. *Chiropotes satanas* *Lagothrix cana cana* *Lagothrix lagotricha lagotricha* *Lagothrix lagotricha poeppigii* *Leontopithecus chrysomelas* *Leontopithecus rosalia* *Mico humeralifer* *Pithecia monachus* *Pithecia irrorata* *Pithecia pithecia* *Saguinus martinsi martinsi* *Saguinus martinsi ochraceous* *Saguinus midas niger* *Saguinus midas* *Saimiri* sp. *Saimiri sciureus* *Saimiri sciureus sciureus* *Saimiri sciureus boliviensis* *Saimiri ustus* *Sapajus apella apella* *Sapajus apella macrocephalus* *Sapajus robustus* *Sapajus xanthosternos*	French Guyana Brazil Venezuela	*Plasmodium brasilianum*	Invasive	Blood smears Conventional microscopy (GIEMSA) PCR ELISA	[14,15,16,17,18,19,20]
*Alouatta guariba clamitans* *Cebus* sp. *Sapajus robustus* *Sapajus xanthosternos*	Brazil	*Plasmodium simium*	Invasive Non-Invasive	Blood smears PCR PCR from faecal samples Nested-PCR	[18,21,22,23]
*Alouatta caraya* *Alouatta guariba* *Alouatta puruensis* *Alouatta seniculus macconnelli* *Ateles chamek* *Callicebus bruneus* *Lagothrix cana cana* *Sapajus apella*	Brazil French Guyana	*Plasmodium falciparum*	Invasive	ELISA IFA PCR	[14,45]

## 2. Results

A total of 26 faecal DNA samples were analysed from captive and free-ranging NHP, in the Western Amazon region of Ecuador. After DNA extraction and using a pan-*Plasmodium* PCR, one positive sample was obtained from an adult female *Leontocebus lagonotus* (representing 3.85%). This animal came from a rescue centre in Pastaza. As with the other rescue centres in Ecuador, most NHP from this rescue centre have been donated by families or confiscated by the police during roadside checks, with this individual’s information being uncertain.

**Figure 1:**
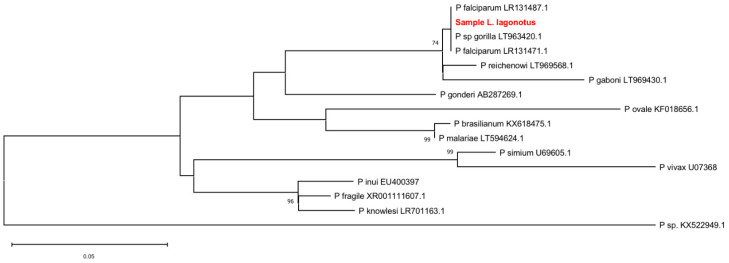
Evolutionary relationships of the *Leontocebus lagonotus* isolate described in this study (bolded and highlighted in red) compared to representative members of the Plasmodium genus. The tree is based on the maximum-likelihood phylogeny of the partial small subunit ribosomal RNA gene. The phylogenetic analysis was performed using the Tamura 3-parameter substitution model implemented in MEGA X. Bootstrap percentages > 70% (1000 resamplings) are indicated at the nodes. GenBank accession numbers are indicated for each strain. The scale bar indicates nucleotide substitutions per site.

We sequenced the amplicon and then aligned it with sequences from other *Plasmodium sp.* species available in the GenBank. The maximum-likelihood phylogeny (ML) tree (Figure 1) yielded a topology with the different *Plasmodium* species clades and an internal (derivated) monophyletic clade comprised by *P. falciparum* including the sequence obtained from the *Leontocebus lagonotus* plus *P. reichenowi* sequences and *P. gaboni*. The ML recovered a clear reciprocal monophyly between *falciparum* and *reichenowi* as sister groups both with a strong support group by bootstrapping values of 74%. The arrangements of other sister clades were recovered: *P. simium + P. vivax*; *P. inui + (P. fragile + P. knowlesi)*; *P. ovale* + (*P. brasilianum + P. malariae*). All sister groups were strongly supported by bootstrapping values of above 96%. In the analysis performed, the sequence of the amplified *Plasmodium* from the NHP *L. lagonotus*’ faeces is located within the *P. falciparum* clade, supporting its molecular identification with this species in the phylogenetic species sense.

## 3. Discussion

DNA from only 26 of the 109 faecal samples collected from 109 NHP was analysed because the quality of the DNA from the other 83 samples was insufficient for the detection of *Plasmodium*, possibly due to the presence of some inhibitors that prevent the amplification of DNA. We detected *Plasmodium falciparum* in a faecal sample from *Leontocebus lagonotus* (3.85%), contributing to the parasite ecology of NW NHP. The percentage of detection is not high, but it is in line with previous published results in scientific literature in Latin America (e.g., [23]).

Malaria parasites display host specificity [47], such as avian malaria in birds [48,49], and malaria in apes [50]. However, it is estimated that the diversification of *Plasmodium* was rapid 16–24 million years ago (MYA) or 26–38 MYA, which differs from the divergence times of the hosts (reptiles to mammals) 75–310 MYA. This difference in the divergence times between parasites and hosts suggests that there has been no co-divergence with the hosts [51]. In primates, two groups of *Plasmodium* have been identified. The first includes the species *P. malariae/P. ovale/P. hylobatid*, which infect Old World primates. The second group includes the species *P. falciparum/P. reichenowi*, which infect humans and NHP. In NW monkeys, *P. brasilianum* and *Plasmodium simium* are known to be the cause of primate malaria. *Plasmodium falciparum* is known to be a cause of human malaria, a result of a recent cross-species transmission of a parasite between gorillas and humans [52]. However, *P. falciparum* is also known to naturally infect at least eight species of NW NHP [14,45]. Our sample is located in the clade of *P. falciparum*, and close to *P. recheinowi*. This relationship was already observed in previous studies [51,53,54]. It has been reported that *Plasmodium falciparum* and *P. reichenowi* are part of a monophyletic clade [55]. 

Non-human primates in captivity from this study are in close contact with care takers and tourists, which may increase the possibility of parasite transmission [56]. However, the origin of these captive animals is unknown. This assumption makes us wonder whether the infection occurred in a sylvatic environment or whether it occurred during the captive period of this NHP. If it occurred during the sylvatic environment, the transmission could be the result of a natural infection via mosquitoes. *Leontocebus lagonotus* is known to be widely distributed in Western Amazonia in Ecuador, i.e., in Pastaza and Morona Santiago provinces [57], which according to the Center for Disease Control and Prevention (CDC) [58], are areas of malaria (*Plasmodium vivax* and *Plasmodium falciparum*). In 2019, Pastaza and Morona Santiago where the two provinces with the highest number of malaria (*Plasmodium falciparum* and *Plasmodium vivax*) cases in humans [59]. If it occurred during the captive period, further studies should be carried out to determine if the vector responsible for the human–NHP transmission is present in this region because there are no reports of the vector in the area. 

## 4. Materials and Methods

### 4.1. Study Sites and Sampling

**Figure 2:**
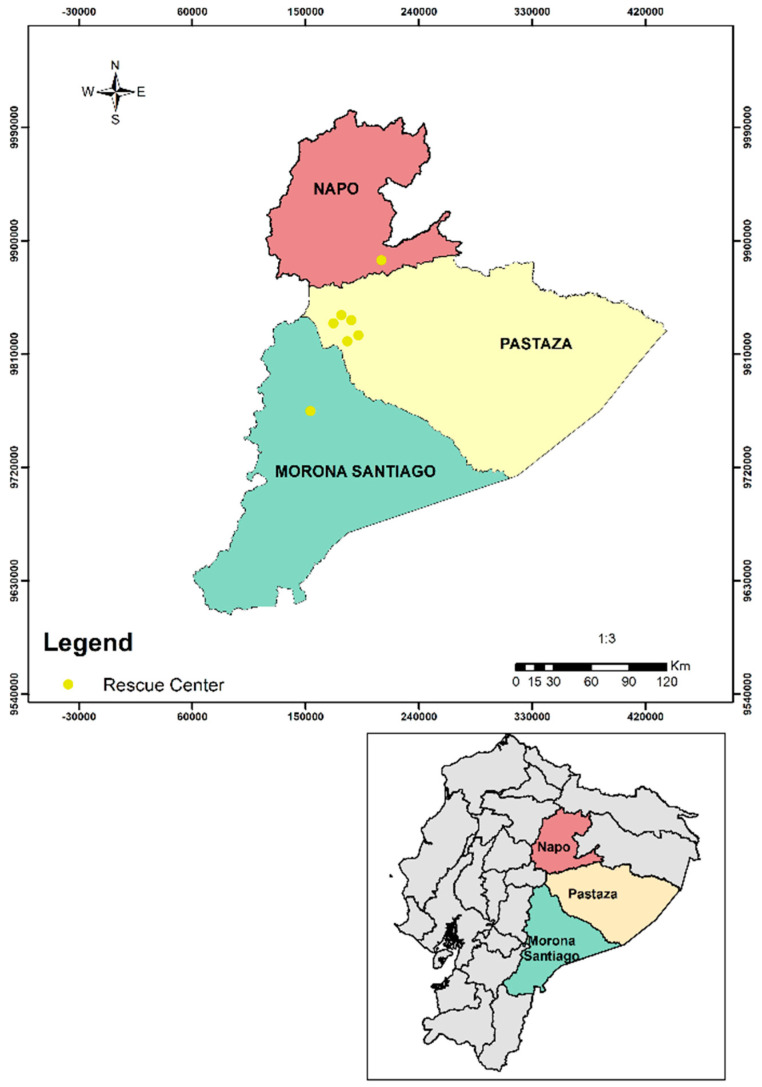
Rescue centres surveyed in the Amazon region of Ecuador.

**Figure 3:**
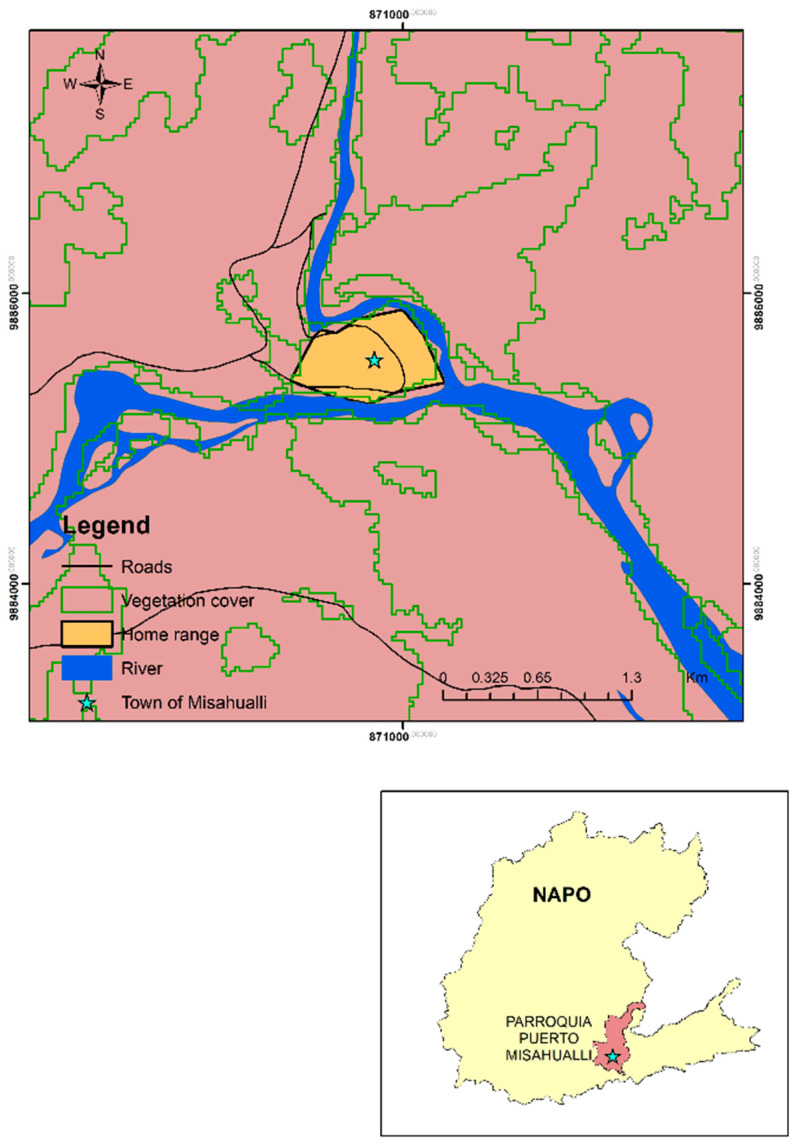
Location of free-ranging population of *Cebus yuracus* surveyed in the Amazon region of Ecuador.

This study was performed in Puyo (Pastaza), Tena (Napo), and Macas (Morona Santiago), three cities in the Western Amazon region (Figure 2). We examined *Plasmodium* spp. in two populations, one captive and one free-ranging. The captive population was studied in wildlife refuges. Most NHP from wildlife refuges had been donated by families or confiscated by the police during roadside checks. The free-ranging population lived in the small town of Misahualli (Tena, Napo) (1°2′7.0″ S, 77°39′59.4″ W) (Figure 3).

Faecal samples are an important source of information about pathogens (viruses, prokaryotes, or eukaryotes) that infect primate species. The analysis of molecular faecal samples offers a non-invasive option that becomes a valid alternative to the traditional sampling methods (blood and tissue samples) of primates [60,61,62,63,64,65,66,67,68,69,70,71,72,73,74,75,76,77]. Studies in conservation genetics have used faecal samples for DNA extraction prioritizing species that are in some category of protection or threat [78,79]. In this study, we collected 109 faecal samples from 109 NHP. However, the DNA quality was not good enough on 83 samples to detect *Plasmodium*. Indeed, we analysed faecal samples (n = 26) from nine species of captive (n = 19) and free-ranging (n = 7) NHPs (Table 2), between 2011 and 2019, in the Western Amazon region of Ecuador from Proyecto Primates Ecuador. Individuals were followed daily from 8 a.m. to 6 p.m. and we collected the samples immediately after defecation to avoid getting confused with samples from other species. All animals were individually identified to facilitate the results analysis. For the molecular analyses, samples were stored in 50 mL Falcon tubes in 99% alcohol at −20 °C to prevent the degradation of DNA. In addition, 600 µL of faeces suspension (1:3; 1 part of faecal sample and 3 parts of ethanol 96–100%) was centrifuged for 2 min at 239 g and the pellet was washed with 1 mL of PBS Buffer (Oxoid, Hampshire, England). This solution (pellet +PBS) was centrifugated for 5 min and the supernatant was discarded. This washing step was repeated three times. Next, the pellet was re-suspended in 600 µL of 2% PVPP (polyvinylpolypyrolidone—Sigma), and frozen overnight at −20 °C to facilitate the capture of phenols in the sample. DNA extraction was performed twice on different days using the QIAamp Stool FAST Mini Kit (Qiagen GmbH, Hilden, Germany) following the manufacturer’s instructions. To prevent cross-contamination, sample preparation, DNA extraction, and the PCR were performed in completely different and separated rooms. Furthermore, the master mix was assembled in a DNA-free room. Ultraviolet light sterilization was performed before and after each procedure. The PCR was carried out using primers targeting the small subunit of 18S ribosomal RNA. The primers used were described by dos Santos et al. [80] (Table 3).

The molecular identification was performed in two reactions (nested PCR) as described by [80], with adaptations. The amplification in the first reaction consisted of 95 °C for 5 min, 95 °C for 30 seg; 50 cycles of 55 °C for 30 seg, 72 °C for 1 min; and a final extension step at 72 °C for 5 min, with a product of 600 bp. In addition, the amplification in the second reaction consisted of an initial denaturation at 95 °C for 5 min, 95 °C for 30 seg; 50 cycles of 58 °C for 30 seg and 72 °C for 1 min; and a final extension at 72 °C for 5 min. The products of the second reaction (240 bp) were observed using the electrophoresis of an agarose gel under UV light. Amplicons were cut, extracted using NucleoSpin gel and the PCR clean-up kit (Macherey-Nagel, Düren, Germany) and sequenced (Sanger sequencing) by Eurofins (Hamburg, Germany). Every PCR reaction contained a negative and a positive control. Sterile filtered pipette tips were used in all stages of the methodology to prevent contamination. We changed the pipette tip after each sample to avoid false-positive reactions/cross-contamination and all laboratory consumables were not reused.

A positive control for *Plasmodium* spp. was obtained using DNA extracted from the spleen of a Belgian blackbird (*Turdus merula*) collected in 2018 [81,82].

### 4.2. Molecular Identification 

The sequence was uploaded to GenBank under the accession number submission MZ156589. Sequence reconstruction was performed using Assembler by MacVector software 17.5.5. The first sequence identity was confirmed by BLAST in NCBI resources. A total of 15 sequences from 14 species of *Plasmodium* were retrieved from GenBank and included as sister groups in order to obtain a wide geographic diversity and taxonomic representation [83] (Table 4). DNA sequences were aligned using MacVector 17.5.5 [84] by the ClustalW algorithm with high gap creation and extension penalties by 30.0 and 10.0, respectively, searching for a strong positional homology. The evolutionary history was inferred by using the maximum likelihood method and the Tamura 3-parameter model. The tree with the highest log likelihood (−932.64) is shown. A discrete Gamma distribution was used to model evolutionary rate differences among sites (five categories (+G, parameter = 0.3352)). The tree was drawn to scale, with branch lengths measured in the number of substitutions per site. This analysis involved 16 nucleotide sequences. There was a total of 241 positions in the final dataset. Evolutionary analyses were conducted in MEGA X.

The robustness for all the analyses was estimated using bootstrapping with 1000 pseudoreplicates and shown in percentage.

## 5. Conclusions

The results of this study provide evidence of *Plasmodium falciparum* in a species of NW NHP, and the potential risk of zoonotic malaria transmission. The present study, by identifying the presence of the parasite in NHP, suggests the need to promote continuous and systematic diagnoses and monitoring of malaria in these animals. In addition, wildlife trafficking and management should be incorporated into public health policies for the prevention of malaria as an emerging zoonotic disease.

## Figures and Tables

**Table 2:** Non-human primate species sampled in the Ecuadorian Amazon.

Habitat Settings	Non-Human Primate Species	n	Sex		Age		
			Male	Female	Adult	Subadult	Juvenile
Captive	*Alouatta seniculus*	4	0	4	1	2	1
	*Ateles belzebuth*	1	1	0	1	0	0
	*Callicebus lucifer*	1	1	0	1	0	0
	*Cebuella pygmaea*	1	1	0	1	0	0
	*Cebus yuracus*	2	1	1	1	1	0
	*Lagothrix lagotricha*	2	0	2	1	0	1
	*Leontocebus fuscicollis*	3	2	1	2	1	0
	*Saimiri sciureus*	3	2	1	1	1	1
	*Sapajus apella*	2	1	1	1	0	1
Free ranging	*Cebus yuracus*	7	5	2	4	1	2

**Table 3:** Sequences of the primers.

Reaction	Primer	Oligonucleotide Sequence	
First reaction	rPLU1	5′-TCAAAGATTAAGCCATGCAAGTGA 3′	forward
	rPLU6R	5′-CGTTTTAACTGCAACAATTTTAA-3′	reverse
Second Reaction	rPLU3	5′-TTTTTATAAGGATAACTACGGAAAAGCTGT-3′	forward
	rPLU4	5′-TACCCGTCATAGCCATGTTAGGCCAATACC-3′	reverse

**Table 4:** GenBank accession numbers of *Plasmodium* species sequences.

Plasmodium Species	ID Genbank	Host	Country
*Plasmodium* sp.	LT963420.1	*Gorilla* sp.	Unknown
*P. falciparum*	LR131487.1	Unknown (Genome assembly)	Unknown (Genome assembly)
*P. falciparum*	MZ156589	*Leontocebus lagonotus*	Ecuador
*P. falciparum*	LR131471.1	Unknown (Genome assembly)	Unknown (Genome assembly)
*P. reichenowi*	LT969568.1	Unknown (Genome assembly)	Unknown (Genome assembly)
*P. gaboni*	LT969430.1	Unknown (Genome assembly)	Unknown (Genome assembly)
*P. gonderi*	AB287269.1	*Cercocebus atys*	Central Africa
*P. ovale*	KF018656.1	*Homo sapiens*	China
*P. brasillianum*	KX618475.1	*Sapajus flavius*	Brazil
*P. malariae*	LT594624.1	Unknown (Genome assembly)	Unknown (Genome assembly)
*P. fragile*	XR001111607.1	Unknown	Unknown
*P. inui*	EU400397	*Macaca fascicularis*	Thailand
*P. simium*	U69605.1	*Saimiris sciureus*	Colombia
*P. vivax*	U07368	Unknown	CDC Strain
*Plamodium* sp.	KX522949.1	*Anopheles nuneztovari*	Brazil
*P. knowlesi*	LR701163.1	Unknown (Genome assembly)	Unknown (Genome assembly)

## Data Availability

The data that support the findings of this study are available from the corresponding author upon reasonable request.
